# Supplementary material for: Social drivers and pediatric injury outcomes in Northern Tanzania: A prospective pediatric injury registry secondary analysis
Source: PLOS Glob Public Health. 2026 Feb 11;6(2):e0005236. doi: 10.1371/journal.pgph.0005236 (PMC12893584; doi:10.1371/journal.pgph.0005236)
Supplement: S1 File — Additional information regarding the pediatric injury registry data collected with SDH factors highlighted. (PDF) [file pgph.0005236.s001.pdf]

Research Assistant: ________ Pt Registry ID#:_____________

# KCMC Pediatric Clinical Trauma Registry

**Patient Dead on Arrival?** ☐No ☐Yes (Demographics only)

## DEMOGRAPHICS

**Where patient lives:** ☐ Moshi Urban ☐Moshi Rural ☐ Other: ___________________

**Patient age:** ______ years ______ months______ days

**Patient sex:** ☐Male ☐Female

**Patient Years Education:** ______ ☐ Unable to determine

**Caregiver Years Education:** ______ ☐ Unable to determine

**Marital status of mother:** ☐ Single ☐ Married ☐ Partner, not married ☐ Widowed ☐ Separated ☐ Died

**Employment of mother:** ☐ Student ☐ Unemployed ☐ Professional ☐ Skilled employment ☐ Self-employed ☐ Farmer ☐ Died ☐ Other:______________________

**Employment of father:** ☐ Student ☐ Unemployed ☐ Professional ☐ Skilled employment ☐ Self-employed ☐ Farmer ☐ Died ☐ Other:______________________

**Patient lives with:** ☐ Single parent ☐ Both parents ☐ Grandparent/Aunt/Uncle (circle) ☐ Other:______________________

**Number of children living in the home:** ☐ 1 (patient) ☐ 2-3 ☐ 4-5 ☐ 6-7 ☐ >7

**Tribe:** ☐ Chagga ☐ Sambaa ☐ Masai ☐ Pare ☐ Sukuma ☐ Iraq ☐ Nyaturu ☐ Mmeru ☐ Muha ☐ Other: ______________

**Type of payment:**

☐ None and abscond ☐ Personal/Family Cash

☐ Nat'l Health Insurance ☐ Hospital exemption (determined at discharge)

☐ Other: ______________________

**If Personal/Family Cash, how will you pay:**

☐ Personal cash only ☐ Contributions from other sources

**Within the past 12 months, have you or your children NOT had enough money for food?**

☐ No, 3 meals per day most days

☐ Yes, no meals per day most days

☐ Yes, 1 meal per day most days

☐ Yes, 2 meals per day most days

☐ Prefer not to answer

**What kind of food are you mostly eating at home (meat, rice, ugali, etc)?** _________________________

## MEDICAL HISTORY

**Malnutrition** (parent report or noted in health booklet):

☐ No

☐ Yes

☐ Don't know

**Asthma** (parent report or noted in health booklet):

☐No

☐Yes

☐ Don't know

**Any other (past) medical condition:**

☐No ☐ Yes ______________________

**Any prior TBI?:** ☐No ☐ Yes

**HIV Status:** ☐ Tested and negative (-) ☐ Never tested ☐ Positive ☐ Unknown ↓

**Prescribed Medications:**

| Medication name | Dose | Frequency |
|---|---|---|
| 1 | | ☐ 1/d ☐ 2/d ☐ 3/d ☐ 4/d ☐ 5/d ☐ as needed |
| 2 | | ☐ 1/d ☐ 2/d ☐ 3/d ☐ 4/d ☐ 5/d ☐ as needed |
| 3 | | ☐ 1/d ☐ 2/d ☐ 3/d ☐ 4/d ☐ 5/d ☐ as needed |
| 4 | | ☐ 1/d ☐ 2/d ☐ 3/d ☐ 4/d ☐ 5/d ☐ as needed |
| 5 | | ☐ 1/d ☐ 2/d ☐ 3/d ☐ 4/d ☐ 5/d ☐ as needed |
| 6 | | ☐ 1/d ☐ 2/d ☐ 3/d ☐ 4/d ☐ 5/d ☐ as needed |

## ACUTE INJURY INFORMATION:

Date of Injury: (dd/mm/20yy) __ __/__ __/20__ __

Injury time: (24 hr) __ __:__ __

Date of Arrival to KCMC: (dd/mm/20yy) ____/____/20____

Arrival time: (24 hr) __ __:__ __

**Mechanism of arrival:** ☐ Ambulance from other hospital ☐ Private car ☐ Bajaji ☐ Police car ☐ Private Motorcycle ☐ Boda boda ☐ Taxi ☐ Walking ☐ Unknown ☐ Other: __________________

**First health center treated at:**

KCMC? ☐ Yes ☐ No →

☐ Hai ☐ Same ☐ Kilema ☐ Kibosho

☐ Faraja ☐ Mawenzi ☐ Siha ☐ St. Joseph

☐ Huruma ☐ TPC ☐ Marangu ☐ Mwanga

☐ Mererani ☐ Usangi

☐ Other:______________________

Arrival date: (dd/mm/yy) __ __/__ __/__ __

Arrival time: (24 hr) __ __:__ __

**Second health center treated at:**

KCMC? ☐ Yes ☐ No →

☐ Hai ☐ Same ☐ Kilema ☐ Kibosho

☐ Faraja ☐ Mawenzi ☐ Siha ☐ St. Joseph

☐ Huruma ☐ TPC ☐ Marangu ☐ Mwanga

☐ Mererani ☐ Usangi

☐ Other:______________________

**Third health center treated at:**

KCMC? ☐ Yes ☐ No →

☐ Hai ☐ Same ☐ Kilema ☐ Kibosho

☐ Faraja ☐ Mawenzi ☐ Siha ☐ St. Joseph

☐ Huruma ☐ TPC ☐ Marangu ☐ Mwanga

☐ Mererani ☐ Usangi

# KCMC Pediatric Clinical Trauma Registry

☐ Other:______________________

**Substance Use Within 6 Hours of Injury:**

☐ Unknown ☐ None ☐ Yes →

→ **Alcohol** ☐ No ☐ Yes

→ **Other substance** ☐ No ☐ Yes

→ **Substance use determined by:**

Self-report ☐ No ☐ Yes

Breathalyzer >0.0 ☐ No ☐ Yes

Clinical Exam ☐ No ☐ Yes

**Location of Injuries**:

| | | | | | |
|---|---|---|---|---|---|
| L1. Head? | ☐ No | ☐ Yes | *If yes, Is it severe?* | ☐ No | ☐ Yes |
| L2. Neck? | ☐ No | ☐ Yes | *If yes, Is it severe?* | ☐ No | ☐ Yes |
| L3. Chest? | ☐ No | ☐ Yes | *If yes, Is it severe?* | ☐ No | ☐ Yes |
| L4. Abdomen? | ☐ No | ☐ Yes | *If yes, Is it severe?* | ☐ No | ☐ Yes |
| L5. Back? | ☐ No | ☐ Yes | *If yes, Is it severe?* | ☐ No | ☐ Yes |
| L6. Legs? | ☐ No | ☐ Yes | *If yes, Is it severe?* | ☐ No | ☐ Yes |
| L7. Feet? | ☐ No | ☐ Yes | *If yes, Is it severe?* | ☐ No | ☐ Yes |
| L8. Arms? | ☐ No | ☐ Yes | *If yes, Is it severe?* | ☐ No | ☐ Yes |
| L9. Hands? | ☐ No | ☐ Yes | *If yes, Is it severe?* | ☐ No | ☐ Yes |

**Intention of the Injury**:

☐ Unknown

☐ Unintentional

☐ Intentional→ ☐ Self-Inflicted

☐ Inflicted by other person

☐ Inflicted by other (non-person)

**Mechanism of Injury:**

☐ **Unknown**

☐ **Road Traffic→**

→☐ Driver or ☐Passenger

→*Vehicle type→*

☐ Motorcycle → Helmet ?☐No ☐Yes ☐Unknown

☐ Car ☐ Bajaji

☐ Truck ☐ Dala dala ☐ Bus

→*Airbag deployed*: ☐No ☐Yes ☐ Don't know ☐ N/A

→*Seat belt used*: ☐No ☐Yes ☐ N/A

→*Car seat used*: ☐No ☐Yes ☐ N/A

→*Ejected from vehicle*: ☐No ☐Yes ☐ N/A

→☐ Pedestrian

→☐ Bicycle → *Helmet?* ☐No ☐Yes ☐Unknown

☐ **Blunt Force / Struck / Hit→**

→☐ By person: ☐No ☐Yes

→☐ Object: ☐No ☐Yes

→☐ Animal: ☐No ☐Yes

☐ **Penetrating Trauma**:→ ☐ Gun ☐ Knife ☐ Animal

☐ **Fall→** ☐ Fall from standing

☐ Fall from height: _______meters from ground

☐ **Burn**

☐ **Drowning**

☐ **Suffocation / Choking / Hanging**

☐ **Poisoning / Toxic Exposure**

☐ **Animal Envenomation→** What Animal? ___________

☐ **Other:**_________________________________________

## BASELINE RISK ASSESSMENT:

**Vital Signs on Arrival to ED:**

T _____ RR ______ HR ______ BP ______ / ______

Pulse Ox ______ Pain Level (0-10)_______ MUAC: _____ cm

Height_____cm Weight______kg

Date:(dd/mm/20yy) ____/____/20____ Time: (24 hr) __ __:__ __

**Pupils:** L: ☐normal ☐sluggish ☐ non-reactive ☐ untestable

R: ☐normal ☐sluggish ☐ non-reactive ☐ untestable

**Are pupils even?** ☐ No ☐ Yes

| | *Children >2 years* | *Children <2 years* | |
|---|---|---|---|
| **Eye Opening** (choose one) | *Spontaneously* | *Spontaneously* | *4* |
| | *To Speech* | *To Sound* | *3* |
| | *To Pain* | *To Pain* | *2* |
| | ***None*** | ***None*** | ***1*** |
| **Verbal Response** (choose one) ☐ T (check if intubated) | *Oriented* | *Age-appropriate vocalization, Coos* | *5* |
| | *Confused* | *Cries, Irritable* | *4* |
| | *Inappropriate* | *Cries to Pain* | *3* |
| | *Incomprehensible* | *Moans to Pain* | *2* |
| | ***None*** | ***None*** | ***1*** |
| **Motor Response** (choose one) | *Obeys Commands* | *Spontaneous Movements* | *6* |
| | *Localizes to pain* | *Withdraws to Touch* | *5* |
| | *Withdraws from pain* | *Withdraws from Pain* | *4* |
| | *Flexion to pain* | *Flexion to Pain* | *3* |
| | *Extension to pain* | *Extension to Pain* | *2* |
| | ***None*** | ***None*** | ***1*** |

**Jeraha ambalo mtoto wako ameumia leo ni baya au kubwa kwa kiasi gani?** *(How serious or 'bad' is the injury your child sustained today?)*

☐ Sio kubwa kabisa *(Not at all serious)*

☐ Sio mbaya sana kwa ajali *(Not too bad of an injury)*

☐ Sio kubwa wala sio dogol *(Neutral)*

☐ Mbaya *(Serious)*

☐ Ni mbaya sana na tishio kwa maisha *(Extremely Serious or Life Threatening)*

**Nurses to answer:** Do the patient's injuries require…?

Hospitalization ☐ No ☐ Yes

To go to theatre ☐ No ☐ Yes

or might be life threatening? ☐ No ☐ Yes

# KCMC Pediatric Clinical Trauma Registry

**Document injury:**

If child 11 years-18 years old:

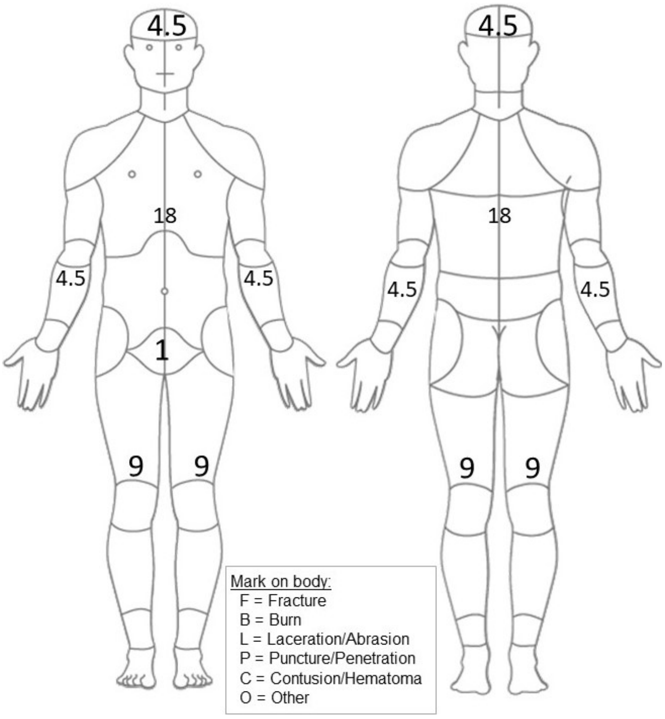


If child 1 day-10 years old:

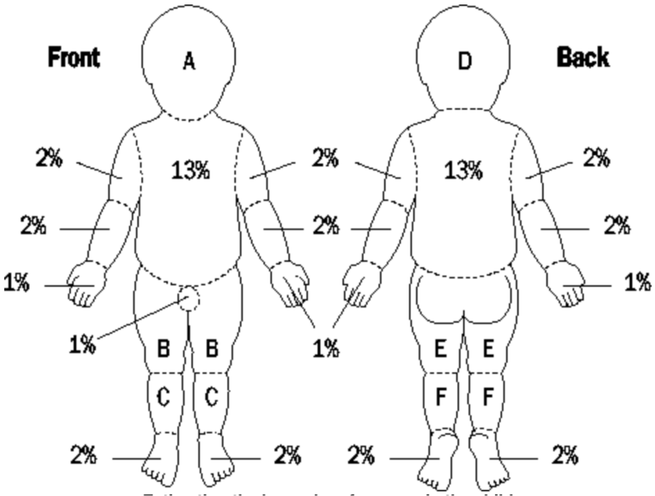


| Area | By age in years | | | |
|---|---|---|---|---|
| | 0 | 1 | 5 | 10 |
| Head (A/D) | 10% | 9% | 7% | 6% |
| Thigh (B/E) | 3% | 3% | 4% | 5% |
| Leg (C/F) | 2% | 3% | 3% | 3% |

Burn? ☐ No ☐ Yes →

If Yes, Total BSA% Burned:__________

# KCMC Pediatric Clinical Trauma Registry

## ED DIAGNOSTICS AND TREATMENT

**Airway & Breathing:**

**Intubation**? ☐ No ☐ Yes→

Date: (dd/mm/20yy) ____/____/20____ Time: (24 hr) __ __:__ __

**Nasopharyngeal airway**? ☐ No ☐ Yes

**Oxygen applied**? ☐ No ☐ Yes

**Circulation:**

**Labs Sent?** ☐ No ☐ Yes

**Fluids Started?** ☐ No ☐ Yes→

Date: (dd/mm/20yy) ____/____/20____ Time: (24 hr) __ __:__ __

**Blood Started?** ☐ No ☐ Yes

**CXR**: ☐ No ☐ Yes

**POCUS/ EFAST:** ☐ No ☐ Yes, if yes→

Date: (dd/mm/20yy) ____/____/20____ Time: (24 hr) __ __:__ __

RUQ: ☐ Normal ☐ Not Done ☐ Abnormal:________________

LUQ: ☐ Normal ☐ Not Done ☐ Abnormal:________________

Cardiac: ☐ Normal ☐ Not Done ☐ Abnormal:________________

Pelvis:☐ Normal ☐ Not Done ☐ Abnormal:________________

Lung: ☐ Normal ☐ Not Done ☐ Abnormal:________________

**Disability:**

**CT head?** ☐ No ☐ Yes

**Spinal Cord Injury?** ☐ No ☐ Yes

**TBI?** ☐ No ☐ Yes

**Vital Signs on Leaving ED**/Arrival to ward/ICU**:**

T _____ RR ______ HR ______ BP ______ / ______

Pulse Ox ______ Pain Level (0-10)________ MUAC: ______ cm

Weight______kg Height_____cm

GCS E___/ V____M___ ☐ T (intubated)

Date:(dd/mm/20yy) ____/____/20____ Time: (24 hr) __ __:__ __

**ED Dispo location**: ☐ Theatre/OR

☐ ICU

☐ Surg 1 ☐ Surg 2

☐ SubICU ☐ Paed

1

☐ Paed 2 ☐ Burn

Unit

☐ Home ☐

Other:___________

**Did the patient's status worsen after the ED?** ☐ No ☐ Yes

## HOSPITAL COURSE

**ICU?** ☐ No *(proceed to "SubICU?")* ☐ Yes (continue) →

Date of Arrival to ICU (dd/mm/20yy): __ __ / __ __ /20__ __

Vital Signs on **Arriving** to ICU**:** (dd/mm/20yy): __ __ / __ __ /20__ __ Time: (24 hr) __ __:__ __

T _____ RR ______ HR ______ BP ______ / ______ Pulse Ox ______

Pain Level (0-10)_______ MUAC: _____ cm Weight______kg GCS E___/ V____☐ T (intubated)/ M____

Date of Discharge from ICU (dd/mm/yy):__ __ / __ __ /20__ __

Vital Signs on **Leaving** ICU**:** (dd/mm/20yy): __ __ / __ __ /20__ __ Time: (24 hr) __ __:__ __

T _____ RR ______ HR ______ BP ______ / ______ Pulse Ox ______

Pain Level (0-10)_______ MUAC: _____ cm Weight______kg GCS E___/ V____☐ T (intubated)/ M____

**SubICU?** ☐ No (proceed to "Needed intubation?") ☐ Yes (continue)

Date of Arrival to SubICU (dd/mm/20yy): __ __ / __ __ /20__ __

Vital Signs on **Arriving** to SubICU**:** (dd/mm/20yy): __ __ / __ __ /20__ __ Time: (24 hr) __ __:__ __

T _____ RR ______ HR ______ BP ______ / ______ Pulse Ox ______

Pain Level (0-10)_______ MUAC: _____ cm Weight______kg GCS E___/ V____☐ T (intubated)/ M____

Date of Discharge from SubICU (dd/mm/yy):__ __ / __ __ /20__ __

Vital Signs on **Leaving** SubICU**:** (dd/mm/20yy): __ __ / __ __ /20__ __ Time: (24 hr) __ __:__ __

T _____ RR ______ HR ______ BP ______ / ______ Pulse Ox ______

Pain Level (0-100)_______ MUAC: _____ cm Weight______kg GCS E___/ V____☐ T (intubated)/ M____

**Needed intubation?** ☐ No ☐ Yes → If yes, where? ☐ EM Dept. ☐ Gen. Surgery ☐ Ortho. Surgery ☐ ICU ☐ Operating room/theatre ☐ Other: __________

→ Intubation: (dd/mm/20yy):__ __ / __ __ /20__ __

→ Extubation: (dd/mm/20yy) ___/___/20___

**ORU (Rehab unit)?** ☐ No ☐ Yes **→ Date to ORU:** (dd/mm/yy) ___/___/___

Research Assistant: ________ Pt Registry ID#:_____________

# KCMC Pediatric Clinical Trauma Registry

**XR Obtained**: ☐ No ☐ Yes → Types: _______________________________________

**Date**: dd/mm/20yy):__ __ / __ __ /20____ Time: (24 hr) __ __:__ __

**Results**: ☐ Pneumothorax ☐ Pleural Fluid ☐ Pulmonary Opacity ☐ Wide mediastinum
☐ Rib Fracture ☐ Pelvic Fracture ☐ C-spine fracture ☐ Extremity Fracture
☐ Other: ____________________

**CT obtained:** ☐ No ☐ Yes→

Date: (dd/mm/20yy):__ __ / __ __ /20____ Time: (24 hr) __ __:__ __

CT Results: ☐ Normal ☐ Abnormal→

| | | | | |
|---|---|---|---|---|
| Subarachnoid Hemorrhage: | ☐ Absent | ☐ Present | ☐ Indeterminant | ☐N/A |
| Subdural Hemorrhage: | ☐ Absent | ☐ Present | ☐ Indeterminant | ☐N/A |
| Epidural Hemorrhage: | ☐ Absent | ☐ Present | ☐ Indeterminant | ☐N/A |
| Other: | ☐ Absent | ☐ Present:_______________________________________________ | | |

## OPERATIVE DATA

**Surgery #1**: ☐ No ☐ Yes→ Date(dd/mm/20yy):__ __ / __ __ /20__ __ Time: (24 hr) __ __:__ __

→ **Indication**:____________________________________________

→ **Procedure**:____________________________________________

→ **Type**: ☐ Orthopedic ☐ General ☐ Neurosurgery ☐ ENT/Otolaryngology ☐ Other

---

**Surgery #2:** ☐ No ☐ Yes→ Date(dd/mm/20yy):__ __ / __ __ /20__ __ Time: (24 hr) __ __:__ __

→ **Indication**:____________________________________________

→ **Procedure**:____________________________________________

→ **Type**: ☐ Orthopedic ☐ General ☐ Neurosurgery ☐ ENT/Otolaryngology) ☐ Other

---

## DIAGNOSTICS LIST (Clinical Impression)

1. Traumatic Brain Injury? ☐ No ☐ Yes→ Type: __________________________________________
2. ______________________________________________________________________________
3. ______________________________________________________________________________
4. ______________________________________________________________________________
5. ______________________________________________________________________________
6. ______________________________________________________________________________

## COMPLICATIONS

| | | |
|---|---|---|
| Pneumonia | ☐ No ☐ Yes→ | ☐ Aspiration ☐ Ventilator Associated ☐ Other |
| Acute renal Injury | ☐ No ☐ Yes | |
| Thrombosis | ☐ No ☐ Yes→ | If yes: ☐ DVT ☐ PE ☐ Fat embolism<br>If yes: ☐ Clinical dx ☐ US ☐ CT |
| Urinary tract infection | ☐ No ☐ Yes | |
| Multiple organ failure secondary to sepsis and shock | ☐ No ☐ Yes | |
| Wound infections | ☐ No ☐ Yes | |
| Wound dehiscence/rupture/burst | ☐ No ☐ Yes | |
| Decubitus Ulcer/ Bed Sore | ☐ No ☐ Yes→ | If yes: ☐ Stage 1 ☐ Stage 2 ☐ Stage 3 ☐ Stage 4 |
| Maternal Psychological Disorder | ☐ No ☐ Yes | If yes: What kind? ______________________ |
| Paternal Psychological Disorder | ☐ No ☐ Yes | If yes: What kind? ______________________ |

**Other Providers involved in care:**

**PT/OT** ☐ No ☐Yes → Date of first service: (dd/mm/yy) ____/____/_____

**Paediatric Consultation** ☐ No ☐Yes→ for: ________________________________________

# KCMC Pediatric Clinical Trauma Registry

**Cardiology Consultation** ☐ No ☐Yes→ for: ________________________________

**Social Worker** ☐ No ☐Yes→ for: __________________________________

**Nutrition** ☐ No ☐Yes→ Date of first service: (dd/mm/yy) ___/___/____

**Psychiatry** ☐ No ☐Yes→ Date of first service: (dd/mm/yy) ___/___/____

**Other:** __________________________ for: _____________________________________

## HOSPITAL DISCHARGE DATA:

**Discharge Vital Signs:**

T _____ RR ______ HR ______ BP ______ / ______ Pulse Ox _______ Pain (0-10)_______

Height_____ cm Weight______kg MUAC: _____ cm GCS E___/ V____☐ T (intubated)/ M____

Vital Signs Date:(dd/mm/20yy) ____/____/20____ Time: (24 hr) __ __:__ __

**Within the past 12 months, have you or your children NOT had enough money for food?**

☐ No, 3 meals per day most days
☐ Yes, no meals per day most days
☐ Yes, 1 meal per day most days
☐ Yes, 2 meals per day most days
☐ Prefer not to answer

**What kind of food are you mostly eating at home (meat, rice, ugali, etc)?** _________________________

**HIV testing during hospitalization:**

☐ Tested and negative (-)
☐ Tested and positive (+)
☐ Tested and result unknown
☐ No documentation of testing

**Was there documentation of HIV testing in the patient chart?**

☐ Yes
☐ No

**Was there documentation of HIV testing in the HTC book?**

☐ Yes
☐ No

**Was there documentation of HIV testing per parent report?**

☐ Yes
☐ No

**Discharge from hospital:** ☐ No, Eloped ☐ No, Death ☐ Yes→ **D/C Date:** (dd/mm/20yy) ____/____/20____ Time: (24 hr) __ __:__ __

**Death in the hospital:** ☐ No ☐ Yes→

**Date of Death:**(dd/mm/20yy) ____/____/20____ Time: (24 hr) __ __:__ __

**Cause of death:** ____________________________________

**Destination** after discharge: ☐ Morgue ☐ Home ☐ Other:____________

# KCMC Pediatric Clinical Trauma Registry

**GOS-E Flow Chart English:**

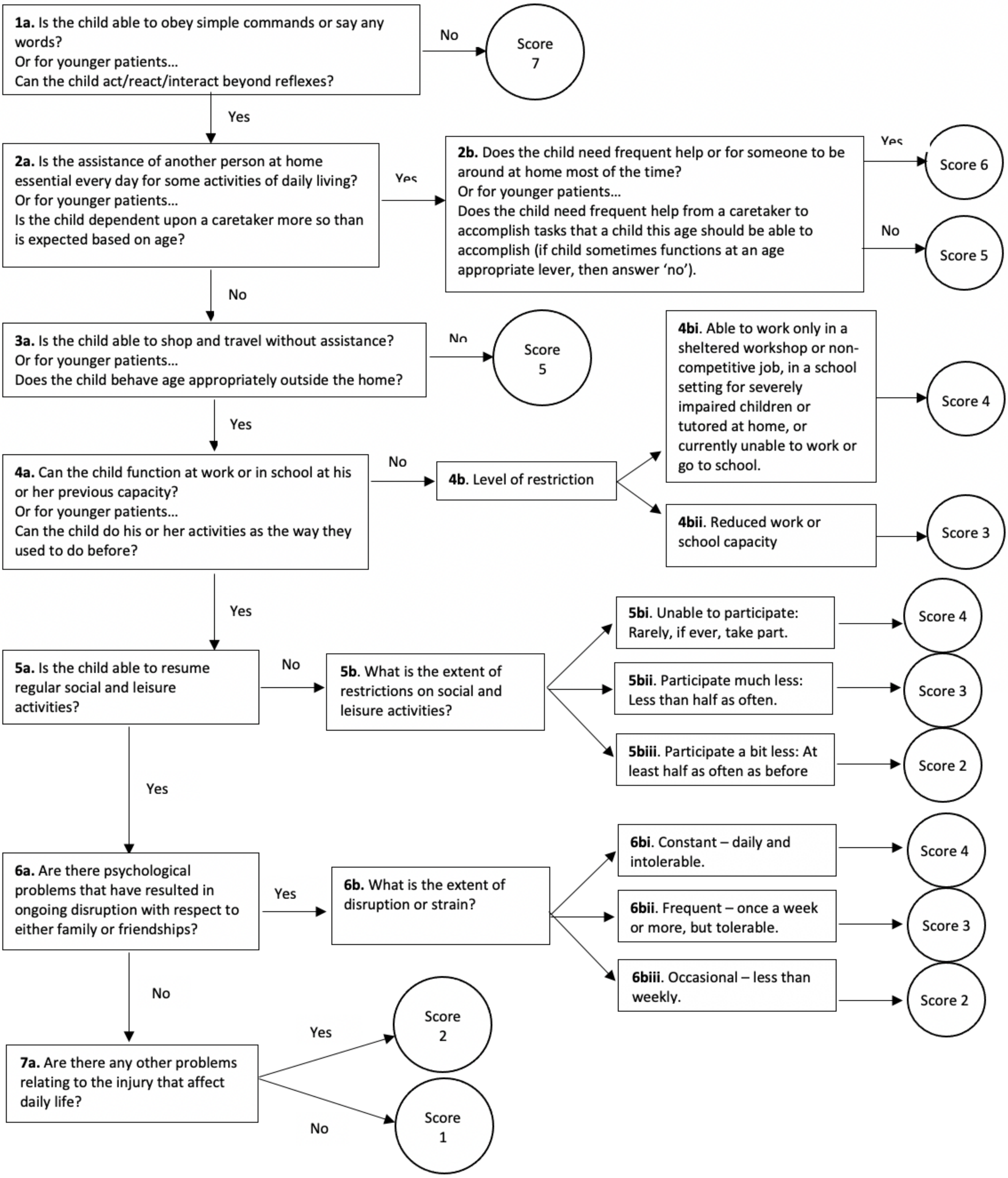

**GOS-E Flow Chart Swahili:**

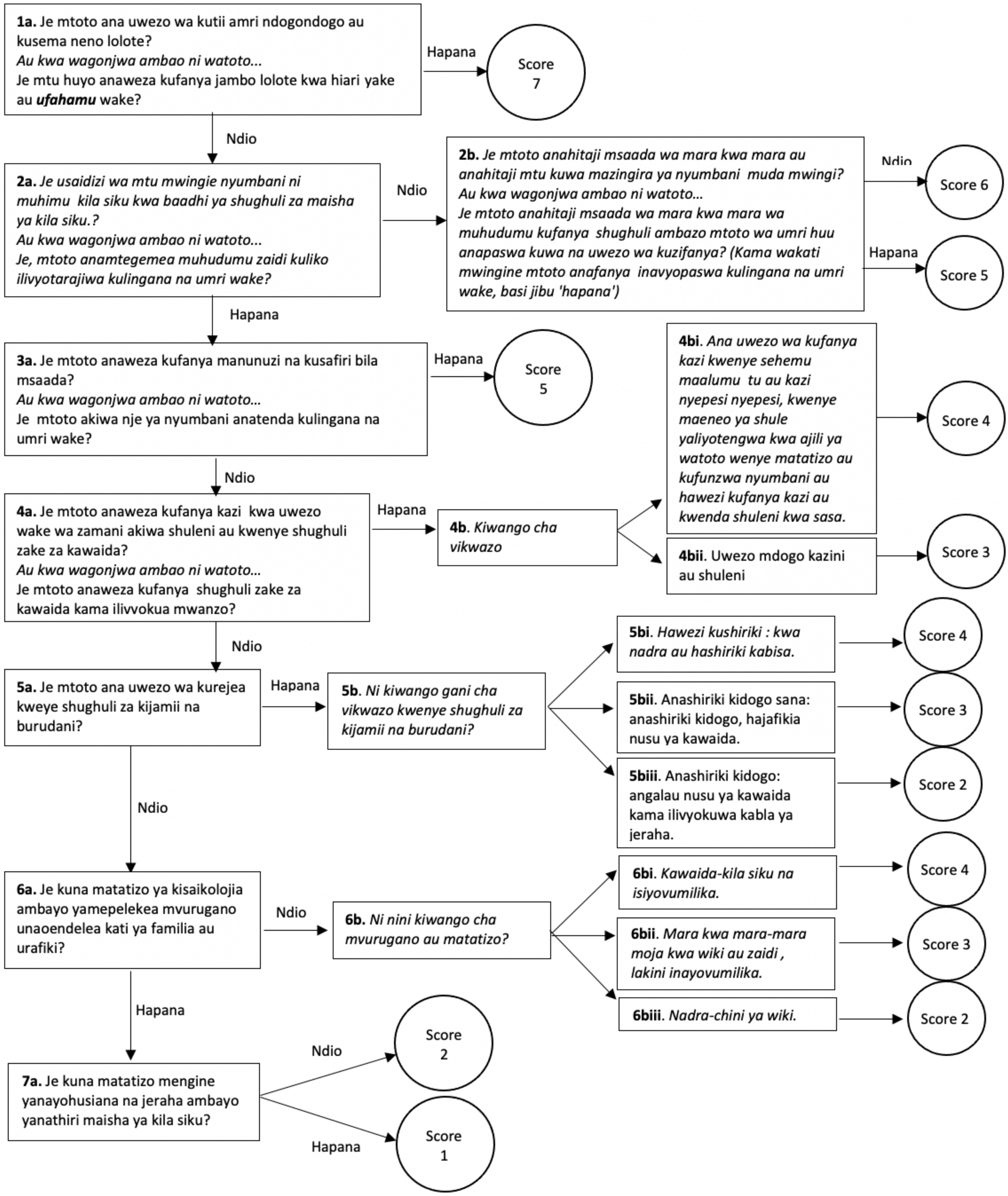

# KCMC Pediatric Clinical Trauma Registry

## GOS-E at Discharge:

| **GOS-E at Discharge**: Use Flow Chart to Score → | |
|---|---|
| ☐ 8 | Death |
| ☐ 7 | Vegetative State (VS) |
| ☐ 6 | Lower Severe Disability (Lower SD) |
| ☐ 5 | Upper Severe Disability (Upper SD) |
| ☐ 4 | Lower Moderate Disability (Lower MD) |
| ☐ 3 | Upper Moderate Disability (Upper MD) |
| ☐ 2 | Lower Good Recovery (Lower GR) |
| ☐ 1 | Upper Good Recovery (Upper GR) |

## GOS-E at 2-week Follow-up:

| **GOS-E at 2-week f/u**: Use Flow Chart to Score → | |
|---|---|
| ☐ 8 | Death |
| ☐ 7 | Vegetative State (VS) |
| ☐ 6 | Lower Severe Disability (Lower SD) |
| ☐ 5 | Upper Severe Disability (Upper SD) |
| ☐ 4 | Lower Moderate Disability (Lower MD) |
| ☐ 3 | Upper Moderate Disability (Upper MD) |
| ☐ 2 | Lower Good Recovery (Lower GR) |
| ☐ 1 | Upper Good Recovery (Upper GR) |

## GOS-E at 3 months Follow-up:

| **GOS-E at 3 months f/u**: Use Flow Chart to Score → | |
|---|---|
| ☐ 8 | Death |
| ☐ 7 | Vegetative State (VS) |
| ☐ 6 | Lower Severe Disability (Lower SD) |
| ☐ 5 | Upper Severe Disability (Upper SD) |
| ☐ 4 | Lower Moderate Disability (Lower MD) |
| ☐ 3 | Upper Moderate Disability (Upper MD) |
| ☐ 2 | Lower Good Recovery (Lower GR) |
| ☐ 1 | Upper Good Recovery (Upper GR) |

## Patient Specific Functional Scale

**Patient Specific Activity Scoring Scheme (Point to one number):**

| **0** | **1** | **2** | **3** | **4** | **5** | **6** | **7** | **8** | **9** | **10** |
|---|---|---|---|---|---|---|---|---|---|---|
| **Hawezi kufanya kabisa** | | | | | | | | | | **Anaweza kufanya kama Ilivyokua kabla ya jeraha** |

| 0 | 1 | 2 | 3 | 4 | 5 | 6 | 7 | 8 | 9 | 10 |
|---|---|---|---|---|---|---|---|---|---|---|
| Unable to perform activity | | | | | | | | | | Able to perform activity at the same level as before injury |

**Patient Specific Functional Scale at Discharge:**

| Activity | Score |
|---|---|
| 1. | |
| 2. | |
| 3. | |
| 4. | |
| 5. | |

**Patient Specific Functional Scale at 2 week Follow-up:**

| Activity | Score |
|---|---|
| 1. | |
| 2. | |
| 3. | |
| 4. | |
| 5. | |

**Patient Specific Functional Scale at 3 months Follow-up:**

| Activity | Score |
|---|---|
| 1. | |
| 2. | |
| 3. | |
| 4. | |
| 5. | |

**--END--**
