# Supplementary material for: Social drivers and pediatric injury outcomes in Northern Tanzania: A prospective pediatric injury registry secondary analysis
Source: PLOS Glob Public Health. 2026 Feb 11;6(2):e0005236. doi: 10.1371/journal.pgph.0005236 (PMC12893584; doi:10.1371/journal.pgph.0005236)
Supplement: Attachment — Submitted filename: PGPH-D-25-02665_Peer Review_PLOS Global Public Health.pdf [file pgph.0005236.s003.pdf]

1. **Title and Abstract**
    a. Study design indicated in the abstract. I suggest adding the study design to the title as well. Comments on the study design are presented later.
    b. **Abstract**
        - It clearly presents the gap, objectives, and methodology.
        - The sociodemographic characteristics (mean age, sex, etc.) of study participants are not mentioned in the results section of the abstract.
        - The abbreviation 'KCMC' is introduced in the abstract without being defined at first mention (line 49).
        - It is not standard practice to cite figures in the abstract. I recommend removing the citation and refining the statement (lines 52-54: "*as illustrated in a Sankey diagram (Figure 1) that depicts the pathways to good (GOS-E Peds ≤2) and poor (GOS-E Peds ≥3) outcomes*"). Instead, you can present the key result directly in the text to ensure the abstract remains clear.
        - The results section lacks numbers (odds ratios, CIs, p-values, etc). Please include these numbers for clarity and interpretability.
2. **Introduction**
    a. The introduction highlights research gaps and objectives, yet it is very brief. More literature could be added to support SDH factors and health outcomes of interest.
3. **Methodology**
    a. Study Design
        i. The study involves the analysis of data collected previously for patient care quality improvement; hence, I suggest refining the design as follows: "This study is a secondary data analysis of prospective data from the pediatric trauma registry at a tertiary referral hospital in Northern Tanzania".
    b. Settings: Line 117 states that the study occurred at KCMC Hospital, which implies that primary data collection was conducted. It would be more accurate to indicate that this study analyzed data from the KCMC Hospital registry, and then proceed with the description of the hospital settings.
    c. Participants: Lines 130-132 imply that the entry of patients into the registry has stopped on January 31, 2024, which is not the case. You should instead clarify that you have analyzed data up to January 31, 2024.
    d. Quantitative procedures section:
        i. This section starts with details related to the registry (including registry on REDCap, etc). This can be added to the section in which you defined the registry.
        ii. There is a redundancy in the details related to data analysis. Please keep such details for the statistical analysis section.
    e. Variables: Morbidity was measured using the Glasgow Outcome Scale Extended Peds (GOS-E Peds). Please include in the text a clear definition of this instrument and its scoring.

SDH domains and how they were measured is not clear in the text. Please clarify.

f. Statistical Methods

i. The version of R software you used does not seem accurate. This is most probably the version of the RStudio. Please double check the R version you have, which typically has a format like (4.3.1; 4.3.2, etc).

ii. Line 159: relative (%) frequencies: put the (%) sign after the word frequencies

4. Results:

a. Descriptive statistics:
Line 186: refine as follows: Morbidity, defined as a poor outcome (GOS-E Peds ≥3), was observed in 38.8% of participants. Also, add the frequency before the percentage.

b. When presenting the logistic regression results, you reported the OR, CI, and p-value for some variables but not for others. Please ensure that all variables are reported consistently.

c. Line 200: “person who patient lives with”: this phrase needs revision.

d. Line 207-208: remove: “as listed in Table 2a and Table 2b” because these variables are also mentioned in Table 3.

e. Lines 213-215: this sentence needs to be refined for clarity:
e.g. Patients who were first seen at KCMC had significantly lower odds of mortality (OR=0.22, 95% CI [0.07-0.56], p=0.05) in univariable LR, but this association did not remain statistically significant in the adjusted model.

f. Line 221: 27% what? Please clarify.

g. Line 225: refine the second part of the sentence for clarity.

h. Lines 231- 239: You should mention significance. Also, numbers are missing. Please modify accordingly.

i. Line 249: Please, remove the sentence “figure 1 illustrates…”.The figure’s content is already defined in the caption.

j. Line 252: the percentage of those who experienced… is a better sentence structure.

k. Line 256: Please remove indicating a disparity and keep the comments for the discussion section.

l. Lines 244–249 read more like “Discussion” rather than “Results”. Please revise this section to maintain the focus on presenting results only.

m. Lines 258-262: The caption is duplicated. Keep only one caption and place it below the figure.

n. Lines 252-256: add frequencies before the percentages n (%).

o. Tables and figures:

i. Table 2a: adding 1 superscript to indicate frequency (%) or median is a bit confusing. I would recommend that you add next to each variable: median (IQR) or n (%). e.g., age (years), median (IQR); sex, n (%), etc.

ii. Add abbreviations in the footnote of each table. e.g., MUAC: mid-upper arm circumference.
iii. Figure 1: What are the health outcomes you are referring to? Please make it clear in the caption.

5. Discussion

a. Line 265: replace “SDH factors” by “data” to make it more general and to avoid repetition: “cross-sectional analysis of *data* in a prospective pediatric trauma registry”.
b. Line 276: Add being: with males *being* at a higher risk …
c. Lines 278-281: You are extensively explaining about ethnicity, which is not reflected in your results. This fits more in the introduction rather than the discussion. You should focus on discussing your results in the discussion.
d. Line 286: replace “can purchase it” with “can obtain coverage” to avoid repetition.
e. Line 308: The sentence structure shows redundancy. This could be formulated as follows: “This could also contribute to patients with more severe conditions presenting in a further deteriorated state upon arrival”.
f. Line 311: You mean lower odds of morbidity and mortality? Please clarify.
g. Lines 329-336: This seems to belong more to the conclusion section.
h. Line 346: who were not transferred *to KCMC*.
i. Line 350: The sentence is too long. Divide it for clarity.

6. Conclusion

Elaborate more on future directions and policy recommendations.
