# Supplementary material for: Social drivers and pediatric injury outcomes in Northern Tanzania: A prospective pediatric injury registry secondary analysis
Source: PLOS Glob Public Health. 2026 Feb 11;6(2):e0005236. doi: 10.1371/journal.pgph.0005236 (PMC12893584; doi:10.1371/journal.pgph.0005236)
Supplement: Attachment — Submitted filename: PGPH-D-25-02665 Review Comments_FMS.pdf [file pgph.0005236.s004.pdf]

**Review of: PGPH-D-25-02665**

**Summary of the study:**

his study is a secondary data analysis of an established registry from a tertiary referral hospital in Northern Tanzania. The authors aim to identify social determinants of health associated with increased morbidity and mortality among pediatric patients who sustained injuries and were transferred to Kilimanjaro Christian Medical Centre.

**Title:**

1- "Pediatric injury outcome" is a very broad term. Please consider rewriting the title into a more specific one. Even though the study is exploratory, "social determinants of health" is too broad.

**Abstract:**

1- It's better to report the multivariable analysis in the abstract rather than reporting the univariable analysis with respect to mortality.
2- What were the variables adjusted for in the "Adjusted Poisson Regression" model for assessing the association with morbidity?
3- Remove the reference to "Figure 1" from the abstract.
4- Under the "Conclusion," it would be better to specify the direction of association between the levels of the independent factor and the outcome. In addition, only report the factors from the multivariable regression in your conclusions.

**Introduction**

1- Line 62 – 67: The link between EXPERIENCING injuries and the mentioned factors is unclear.
2- Line 73 & 74: What are these disadvantages? And how did you relate this to your topic under study?
3- "SDH and pediatric trauma" is a very broad phrase and could be answered by several study designs based on your main objective. Please be more concise.
4- Line 77 & 78: Clarify what you're referring to as health inequality monitoring and keep it within the scope of your study.
5- Line 77 – 80: Understanding the complex interplay between SDH and the health outcomes of injured children doesn't help you identify effective strategies to prevent injuries from happening. To prevent injuries in the first place, you need to study the factors associated with their incidence. In this case, you must approach your study design differently, such as conducting a cohort study or a case-control study. On the

other hand, to prevent the increased severity and negative outcomes of an injury, you focus on the factors associated with post-injury trajectories. Please revise this section accordingly.

6- Line 82: The gap doesn't match your aim.
7- Lines 85 & 86: On what basis were the SDH factors selected?

**Methods**

1- Identify the study as a secondary data analysis study since the registry wasn't created initially to answer the research question under your current submission.
2- Outcomes – mortality and morbidity – should be defined in detail in a separate section, "Study Outcome Variables."
3- Lines 116 – 120 should reflect that data collection for the registry and not this study was conducted at KCMC.
4- It would be better to describe the registry, data collectors' strategies, research assistants following up with patients' care, variables collected, and follow-ups in the main manuscript text and keep the details in the supplementary material.
5- Lines 152 – 155: Move to "Statistical Analysis" section.
6- Under the "Statistical Analysis" section, identify between parentheses the variables' names and measurement scales for each of the categorical and continuous types.
7- You can assess differences at the bivariable level using the Chi-square and Wilcoxon rank sum tests, but not to report adjusted measures of association. Please revise accordingly.
8- Lines 163 – 165: Which models are you referring to? And on what basis did you decide to compare the models?
9- What tools were used to assess independent variables? Validity of the tools? The reader expects to find this information in the Methods without the need to go back to the original data collection methods and details of the registry. More details could be accessed through the reference provided, but such important information on tools is needed under the Methods section.
10- What is your definition of Survivor versus non-Survivor? Time-frame?
11- The definition and classification of Good or Poor outcomes must be included in the Methods section, as this is currently missing from your submission. At present, the first mention appears in the Table and subsequently in the Results. Please revise accordingly.
12- Report on the variables with missing data and the respective percentages.
13- Define the timeframe you're referring to for mortality and morbidity assessments.

14- Did you rely on GOS-E only for assessing morbidity (i.e., specifically TBI)?
15- Which modified Poisson regression was utilized?

**Results**

1- Do not report univariable analysis (i.e., chi-squared test and Wilcoxon rank test) as statistical tests to conclude associations. Please use them for screening potential co-variables and focus on multivariable analysis.
2- In the adjusted analysis, how did you treat the "Mechanism of Injury" groups? How did you enter it in the model, and how did you analyze the different groups?
3- Lines 219 – 221: The age results reporting is unclear. Please revise.
4- The titles of the tables are misleading. Consider revising them by indicating the outcome variable clearly (survivors is misleading), the number of participants, the region(s), and the year(s).
5- Lines 244 – 256: This is exploratory analysis or post-hoc analysis. You should mention this under the methods section and categorize it under a separate section in the Results.
6- Lines 244 – 256: Where are your comparison methods and results?
7- I don't understand the need for a Sankey diagram. The reasoning is unclear to me. It is a visualization tool. How has it solved the problem you're referring to?
8- Don't summarize, interpret, or discuss any of your results/findings in the Results section. Move it to the Discussion (e.g., Our analysis showed; indicating a disparity)

**Discussion**

1- Lines 271 and 272: The significance here should be very specific to your results and not a general conclusion.
2- Lines 274 - 281: The reference to the literature must be cited next to it and not next to your results. In addition, sex and ethnicity are two different factors. Present them in two separate paragraphs or two separate ideas. Provide more examples of how each factor affects, while referencing relevant literature or sources.
3- Lines 283 – 285: Use parallel reporting when comparing with the literature: present exposure and outcome in the same order across studies to facilitate comparison. For example: "Lack of health insurance was associated with increased morbidity and mortality in our study, consistent with prior findings linking lack of insurance to poor health outcomes." Avoid reversing the structure (e.g., "Poor outcomes were previously linked to lack of insurance") as it disrupts the comparative flow, whenever possible.
4- Delve further into the potential mechanisms underlying the observed associations, and avoid broad or generic explanations such as "Several studies have highlighted

the significant role SDH has on the health outcomes of children experiencing injuries in LMICs, 294 noting that poverty was associated with sustaining more severe injuries, having more extended hospital stays, and experiencing poorer outcomes." Instead, emphasize the specific pathways, the how and why SDH influences these outcomes.

5- Lines 298 – 309: Support this finding by presenting the severity status of your study patients and emphasizing this in your Results section.
6- Lines 321 – 327: Such conclusions require path and moderator analysis.

**Conclusions**

This is too general. Please consider grounding it in your specific results to enhance clarity and the reader's understanding of the study's specific significance and relevance.
