# Supplementary material for: Social drivers and pediatric injury outcomes in Northern Tanzania: A prospective pediatric injury registry secondary analysis
Source: PLOS Glob Public Health. 2026 Feb 11;6(2):e0005236. doi: 10.1371/journal.pgph.0005236 (PMC12893584; doi:10.1371/journal.pgph.0005236)
Supplement: Attachment — Submitted filename: Response to Reviewers.pdf [file pgph.0005236.s005.pdf]

BerbeeWalsh Department of Emergency Medicine
UNIVERSITY OF WISCONSIN
SCHOOL OF MEDICINE AND PUBLIC HEALTH

November 12, 2025

Catherine Kyobutungi, MBBS, PhD
Editor-in-Chief
PLOS Global Public Health

Dear Dr. Kyobutungi,

We sincerely thank you for your thoughtful and constructive feedback on our manuscript (PGPH-D-25-02665), *"Exploring the Relationship Between Social Determinants of Health and Pediatric Injury Outcomes at a Northern Tanzania Tertiary Referral Hospital."* We deeply appreciate the time and care taken to review our work. The comments provided were invaluable and have significantly strengthened the clarity, methodological rigor, and policy relevance of our revised manuscript, "*Social Drivers and Pediatric Injury Outcomes in Northern Tanzania: A Prospective Pediatric Injury Registry Secondary Analysis*."

In response, we have substantially revised the paper to address all points raised. Specifically, we:

- Clarified and expanded the **Methods** section to better describe registry enrollment, inclusion criteria, and data quality assurance procedures.
- Reorganized the **Results** section to follow a logical sequence (demographics → univariable results → multivariable results → Sankey visualization).
- Expanded the **Discussion** with subheadings to highlight principal findings, comparisons with prior studies, and policy implications aligned with the **Sustainable Development Goals (SDGs)**.
- Updated the **references (2020–2024)** to reflect recent literature on social determinants of health (SDH) and pediatric injury outcomes in LMICs.
- Revised the **Abstract** for concision and alignment with *PLOS* guidelines.

We also improved formatting, standardized terminology, and added a clearer narrative connecting SDH factors—such as insurance status, food insecurity, and community type—to pediatric injury outcomes.

Below is a summary of the major revisions, followed by detailed, point-by-point responses.

Summary of Major Revisions

Reviewer Comment (summary)	Change Made	Manuscript Location (Line Numbers)
Clarify trauma registry design and data collection.	Expanded Methods section describing registry enrollment, inclusion/exclusion criteria, and variables.	Lines 101–162
Improve organization of Results section.	Reordered to demographics → univariable → multivariable → Sankey diagram visualization.	Lines 201–290
Strengthen Discussion with global context and SDH focus.	Added subheadings, integrated global literature, and connected findings to SDG targets.	Lines 292–386

Reviewer Comment (summary)	Change Made	Manuscript Location (Line Numbers)
Clarify regression modeling and SDH definitions.	Defined SDH variables explicitly and detailed regression approaches.	Lines 128–199
Expand policy implications.	Added "Implications and Future Research" subsection.	Lines 379–386
Update and expand references.	Added new references (2020–2024) relevant to SDH and pediatric injury in LMICs.	Throughout manuscript

---

Editorial Requests
Comment:
"The study offers an important contribution but requires stronger framing around the global relevance of SDH and its implications for LMIC trauma systems."
Response:
Thank you for this valuable suggestion. We expanded the **Introduction** to emphasize the global relevance of SDH and pediatric trauma outcomes, citing recent and relevant literature. We also reframed the **Discussion** to contextualize our findings in LMIC health system strengthening and equity monitoring.

Reviewer #1 Comments and Responses
Comment 1:
"The abstract is lengthy and could be made more concise while retaining key findings."
Response:
We revised the **Abstract** to ensure clarity and brevity while retaining all essential details—study objective, key results, and policy implications. The revised version now aligns with *PLOS Global Public Health*'s word count and structure guidelines.

Comment 2:
"The Methods section should include more details about the trauma registry, enrollment process, and inclusion criteria."
Response:
We appreciate this important suggestion. The **Methods** section now includes expanded details on registry enrollment, data abstraction, and inclusion/exclusion criteria. We clarified that all pediatric trauma patients under 18 years presenting to the ED with acute injuries between November 2020 and January 2024 were enrolled.

Comment 3:
"The Results section feels fragmented; please present findings in a more coherent structure."
Response:
We reorganized the **Results** to improve flow: (1) demographics and injury characteristics, (2) univariable results, (3) multivariable analyses, and (4) Sankey visualization.

Comment 4:
"The Discussion could better connect the findings to international SDH literature."
Response:
We expanded the **Discussion** with new citations linking our results to broader SDH findings in

LMIC trauma research. We highlighted the parallels between Tanzanian outcomes and those seen in other Sub-Saharan African countries.

Comment 5:
"Consider including global health policy implications."
Response:
We added a dedicated subsection titled **"Implications and Future Directions"** that discusses how addressing inequities in insurance access and food security aligns with SDG targets.

Reviewer #2 Comments and Responses
Comment 1:
"The SDH variables (e.g., food insecurity, insurance) need clearer definitions."
Response:
We added explicit operational definitions for each SDH variable in the **Methods**, specifying how food security was measured (daily meal frequency) and how insurance status and community type were categorized.

Comment 2:
"Expand on how rural residence affects injury outcomes."
Response:
We incorporated additional interpretation into the **Discussion**, noting that rural children often face longer prehospital delays, limited transport access, and resource-constrained care, all of which contribute to poorer outcomes.

Comment 3:
"Some Discussion sentences are repetitive; please tighten the text."
Response:
We carefully edited the **Discussion** to remove redundant statements and improve logical progression. This streamlined version emphasizes key points while maintaining interpretive depth.

Comment 4:
"Add study limitations and suggestions for future research."
Response:
We added **Limitations** and **Implications and Future Directions** subsections describing the single-site design, residual confounding, and recommendations for multicenter and qualitative follow-up studies.

Reviewer #3 Comments and Responses
Comment 1:
"Please provide greater detail about data validation and quality assurance in the registry."
Response:
We expanded the **Methods** to describe data verification procedures, including double data entry, routine audits, and resolution of discrepancies by trained research assistants.

Comment 2:
"The Sankey diagram is interesting but could use a clearer explanation."
Response:
We added interpretive text to the **Results** explaining that the Sankey diagram visualizes how

food insecurity and insurance status intersect to influence morbidity trajectories (good vs. poor outcomes).

Comment 3:
“Include more recent references to strengthen context.”
Response:
We updated references throughout the manuscript to include recent literature published from 2020–2025 on SDH and pediatric trauma in LMICs.

Closing Statement
We are deeply grateful to the editor and reviewers for their constructive insights and thoughtful feedback. These revisions have substantially strengthened our manuscript’s clarity, structure, and global relevance. We believe the revised version offers a stronger contribution to understanding the role of social determinants in pediatric injury outcomes in LMICs.

We appreciate the opportunity to revise this work and look forward to your consideration.

Sincerely,

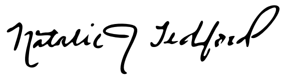

Natalie J Tedford, MD, MScPH, FAAP
University of Wisconsin School of Medicine & Public Health
Assistant Professor (CHS, Emergency Medicine & Pediatrics)
BerbeeWalsh Department of Emergency Medicine
800 University Bay Drive, Suite 300 – 51, Madison, WI 53705
Cell: +1(801) 573-5915
Email: *ntedford@medicine.wisc.edu* or *natalie.j.tedford@gmail.com*
