# Supplementary material for: Social drivers and pediatric injury outcomes in Northern Tanzania: A prospective pediatric injury registry secondary analysis
Source: PLOS Glob Public Health. 2026 Feb 11;6(2):e0005236. doi: 10.1371/journal.pgph.0005236 (PMC12893584; doi:10.1371/journal.pgph.0005236)
Supplement: Attachment — Submitted filename: Re-review FMS_122425.pdf [file pgph.0005236.s006.pdf]

Minor Re-Revision Comments:

1. The definition of "injury" should not be placed under the "Study Participants" section. Please relocate it to the appropriate section.

2. There is inconsistency in your terminology; you use "food security" in some instances and "food insecurity" in others. Please maintain consistent terminology throughout the entire manuscript, focusing on the term that aligns with its role as a predictor.

3. You conducted assumption testing. Its results must be included in the results section, even if only briefly.

4. You reported the results of the modified Poisson regression, but you stated: "AIC was selected from an ordinary logistic regression model compared with a modified Poisson model." Please clarify what you meant by this statement.

5. The titles of the tables are misleading and lack informativeness. Please revise them and use "deaths" or "mortality" instead of "non-survivor."

6. There appears to be an issue with some variables in the reported tables. For example, in Table 3, the adjusted odds ratio (AOR) for mortality regarding the predictor "food insecurity" is listed as 2.62 for the "No" versus "Yes" response. This suggests that "food insecurity" is protective, which is inaccurate. Please revisit this and ensure the categorization and labeling of your variables, including their corresponding reference levels in the analysis, are correct.

7. In the first paragraph of the Discussion section, please replace the phrases "In comparison" and "meaningful relationship."

8. Emphasize more how the limitations of your study might have distorted or affected the measures of association reported in the results.
