# Supplementary material for: Social drivers and pediatric injury outcomes in Northern Tanzania: A prospective pediatric injury registry secondary analysis
Source: PLOS Glob Public Health. 2026 Feb 11;6(2):e0005236. doi: 10.1371/journal.pgph.0005236 (PMC12893584; doi:10.1371/journal.pgph.0005236)
Supplement: Attachment — Submitted filename: Response to Reviewers.docx [file pgph.0005236.s007.docx]

**Response to Reviewers**

**Dear Editor and Reviewers,**

We thank you for the opportunity to revise our manuscript and for the thoughtful, constructive feedback. We have carefully addressed each comment and made substantial revisions to improve the clarity, accuracy, and rigor of our reporting. Below, we provide a point-by-point response to each reviewer’s comment, with specific reference to changes made in the revised manuscript.

**REVIEWER #1 COMMENTS**

**Comment: "In the section of participants characteristics, please indicate what table the reader should refer to in order to verify results."**

**Response:** We have revised the Results section to explicitly reference the appropriate tables. Specifically, we now state: "Participant characteristics stratified by mortality status are presented in Table 1, and characteristics stratified by morbidity outcome are presented in Table 2."

**Location in revised manuscript:** Results section, Participant Characteristics subsection, page 10, Lines 225-226.

**Comment: "In the Mortality analysis section, the P-Value for insurance is reported to be (0.027) which is inaccurate as it reflects the results from table 2 for morbidity outcomes. The actual P-value here is 0.012 (from Table 1). Please make sure to review all the reported values again."**

**Response:** We sincerely apologize for this error. We have carefully reviewed all reported p-values throughout the Results section and corrected this mistake. The p-value for insurance in the mortality analysis now correctly reads p=0.012, consistent with Table 1. All other values have been verified against the corresponding tables.

**Location in revised manuscript:** Results section, Mortality Analysis subsection, page 16, Line 244.

**Comment: "Results reported from Table 3 also require revision in terms of analysis. Report numbers in this section: Food insecurity and lack of insurance both trended toward higher prevalence of poor outcomes, but these associations did not reach statistical significance after controlling for confounders. No consistent differences were observed in caregiver education or household composition after controlling for other SDH variables."**

**Response:** We have revised this section to include specific point estimates and confidence intervals from Table 3.

**Location in revised manuscript:** Results section, Mortality Analysis subsection, page 16, Lines 259-263.

**Comment: "Please review reported results of the Sankey Diagram. The title of Figure 1 should be placed with the figure not separately."**

**Response:** We have reformatted Figure 1 so that the title appears directly with the figure rather than in a separate location. We have also revised the text describing the Sankey diagram to better reflect its exploratory nature and its purpose of illustrating pathways of social vulnerability.

**Location in revised manuscript:** Results section, Exploratory Visualization subsection, page 21 Lines 298-307, Figure 1 legend.

**REVIEWER #3 COMMENTS**

**Comment: "The definition of 'injury' should not be placed under the 'Study Participants' section. Please relocate it to the appropriate section."**

**Response:** We have relocated the definition of "injury" from the Study Participants section to a new subsection titled "Case Definition" within the Methods section, immediately following the Study Setting subsection.

**Location in revised manuscript:** Methods section, Case Definition subsection, page 6, Lines 117-124.

**Comment: "There is inconsistency in your terminology; you use 'food security' in some instances and 'food insecurity' in others. Please maintain consistent terminology throughout the entire manuscript, focusing on the term that aligns with its role as a predictor."**

**Response:** We appreciate this important observation. We have standardized our terminology throughout the manuscript to use "food insecurity" consistently as the predictor variable of interest, in alignment with standard epidemiologic practice. We now clearly define food insecurity in the Methods section and use this term consistently in the Results, Discussion, and Tables.

**Location in revised manuscript:** Throughout manuscript; specifically Methods (SDH Variables subsection, page 7, Lines 154-155), Results (Tables 1-4), and Discussion.

**Comment: "You conducted assumption testing. Its results must be included in the results section, even if only briefly."**

**Response:** We have added a brief statement in the Results section documenting that regression assumptions were tested and satisfied.

**Location in revised manuscript:** Results section, immediately following the Participant Characteristics subsection, page 10-11, Lines 231-235.

**Comment: "You reported the results of the modified Poisson regression, but you stated: 'AIC was selected from an ordinary logistic regression model compared with a modified Poisson model.' Please clarify what you meant by this statement."**

**Response:** We apologize for the confusion in our original wording. We have clarified this statement in the Statistical Analysis section.

**Location in revised manuscript:** Methods section, Statistical Analysis subsection, page 8-9, Lines 180-186.

**Comment: "The titles of the tables are misleading and lack informativeness. Please revise**

**them and use 'deaths' or 'mortality' instead of 'non-survivor.'"**

**Response:** We have revised all table titles to be more informative and to use "mortality" or "death" rather than "non-survivor." The revised titles are:

- Table 1: "Socio-demographic characteristics of participants stratified by in-hospital mortality status"

- Table 2: "Socio-demographic characteristics of participants stratified by morbidity outcome" (unchanged)

- Table 3: "Univariate and multivariate logistic regression models for in-hospital mortality"

- Table 4: "Univariate and multivariate modified Poisson regression models for the prevalence ratio of morbidity/poor outcome (GOS-E Peds ≥3)" (unchanged)

**Location in revised manuscript:** Tables 1-4.

**Comment: "There appears to be an issue with some variables in the reported tables. For example, in Table 3, the adjusted odds ratio (AOR) for mortality regarding the predictor 'food insecurity' is listed as 2.62 for the 'No' versus 'Yes' response. This suggests that 'food insecurity' is protective, which is inaccurate. Please revisit this and ensure the categorization and labeling of your variables, including their corresponding reference levels in the analysis, are correct."**

**Response:** We thank the reviewer for identifying this critical issue. We have carefully reviewed all variable coding and reference categories in our regression models. The paradoxical finding regarding food security reflects the observed data pattern in our cohort: children from food-secure households (able to provide ≥3 meals daily) had higher mortality in adjusted models. This counterintuitive finding likely reflects unmeasured confounding by injury severity, mechanism, or socioeconomic gradients.

We have now:

1. We chose *not* to recode the variable so that "food-insecure" (unable to provide ≥3 meals daily) is the exposure and "food secure" is the reference category, since there is no difference in interpreting the adjusted odds ratio in the direction of this finding.

2. Clearly labeled all reference categories in Tables 3 and 4

3. Added extensive discussion of this paradoxical finding in both the Results and Discussion sections, acknowledging the limitations of our single-item food security measure and the potential for confounding

**Location in revised manuscript:** Tables 3 and 4 (reference categories now clearly labeled); Results section, page 16, Lines 259-263; Discussion section, Food Security and Child Vulnerability subsection, pages 23-25, Lines 367-403; Limitations section, page 26, Lines 440-442.

**Comment: "In the first paragraph of the Discussion section, please replace the phrases 'In comparison' and 'meaningful relationship.'"**

**Response:** We have revised the first paragraph of the Discussion to remove these phrases and use more precise language.

**Location in revised manuscript:** Discussion section, first paragraph, page 21-22, Lines 320-329.

**Comment: "Emphasize more how the limitations of your study might have distorted or affected the measures of association reported in the results."**

**Response:** We have substantially expanded the Limitations section to explicitly discuss how each limitation may have distorted our measures of association. Specifically, we now discuss:

1. How selection and survival bias from the single-center tertiary referral design likely underestimated mortality risk factors

2. How the absence of validated injury severity scores prevented adequate adjustment for confounding and may have produced spurious associations (e.g., the food security paradox)

3. How measurement error in self-reported SDH data and use of a single-item food security measure may have attenuated true associations

4. How the cross-sectional design and residual confounding limit causal inference

The revised Limitations section is now presented as a condensed single-paragraph format per the previous feedback, with explicit discussion of how each limitation affects the interpretation of our findings.

**Location in revised manuscript:** Discussion section, Limitations subsection, page 26-27, Lines 432-449.

**ADDITIONAL REVISIONS**

Beyond the specific reviewer comments, we have made the following additional improvements:

1. **Figure quality:** We have processed all figures through the PLOS NAAS tool as recommended and are submitting the NAAS-processed TIFF files with this revision.

2. **Changes to the Reference list:** In accordance with journal requirements, we have reviewed and updated our reference list. We confirm that no retracted articles were cited in either the original or revised manuscript. The following changes were made:

- **References Removed (n=5):** The following references from the original submission were removed as they were replaced with more current or directly relevant literature:

1. Ruiz-Casares M. "Unintentional childhood injuries in sub-Saharan Africa: an overview of risk and protective factors." *J Health Care Poor Underserved.* 2009. — *Removed as the content was adequately covered by other retained references on pediatric injury in LMICs.*

2. Marmot M, Health CoSDo. "Achieving health equity: from root causes to fair outcomes." *Lancet.* 2007. — *Removed as the WHO Commission on Social Determinants of Health report (Reference 5) provides more comprehensive coverage of this framework.*

3. Simkiss DE, et al. "Childhood disability and socio-economic circumstances in low and middle income countries: systematic review." *BMC Pediatr.* 2011. — *Removed and replaced with more recent systematic reviews on SDH and child health outcomes.*

4. Mostert S, et al. "Influence of health-insurance access and hospital retention policies on childhood cancer treatment in Kenya." *Pediatr Blood Cancer.* 2014. — *Removed as it focused on oncology rather than trauma; replaced with references more directly relevant to injury outcomes and insurance status in LMICs.*

5. Hyder AA, Razzak JA. "The challenges of injuries and trauma in Pakistan: an opportunity for concerted action." *Public Health.* 2013. — *Removed as it was geographically less relevant to the Tanzanian context; replaced with references from sub-Saharan Africa.*

- **References Added (n=6):** The following references were added to strengthen the manuscript with more current evidence:

1. **Heidkamp** **RA.** “Mobilising evidence, data, and resources to achieve global maternal and child undernutrition targets and the Sustainable Development Goals: an agenda for action.” *Lancet*. 2021;397(10282):1400-18. – *Added to provide authoritative linkage between our findings and the Sustainable Development Goals framework; particularly SDG 2 (Zero Hunger), SDG 3 (Good Health and Wellbeing), and SDG 10 (Reduced Inequalities). This Lancet Commission paper establishes the evidence base for integrating nutrition, health equity, and child health monitoring systems—directly relevant to our recommendation for SDH-informed injury registries in LMICs.*

2. **Beyene SD.** "The impact of food insecurity on health outcomes: empirical evidence from sub-Saharan African countries." *BMC Public Health.* 2023;23(1):338. — *Added to provide current, regionally relevant evidence on food insecurity and health outcomes in sub-Saharan Africa, directly supporting our discussion of the paradoxical food security findings.*

3. **Gallegos D.** "Effects of Food and Nutrition Insecurity on Global Health." *N Engl J Med.* 2025;392(7):686-97. — *Added to provide the most current comprehensive review of food insecurity's effects on health outcomes globally, including discussion of methodological challenges in measuring food insecurity and interpreting paradoxical findings.*

4. **Mahboob A, et al.** "Childhood unintentional injury: The impact of family income, education level, occupation status, and other measures of socioeconomic status. A systematic review." *Paediatr Child Health.* 2021;26(1):e39-e45. — *Added to provide systematic review evidence on socioeconomic determinants of childhood injury.*

5. **Bou-Karroum L, et al.** "Financing for equity for women's, children's and adolescents' health in low- and middle-income countries: A scoping review." *PLOS Glob Public Health.* 2024;4(9):e0003573. — *Added to provide current evidence on health financing equity in LMICs, supporting our discussion of insurance access.*

6. **GBD 2021 Diseases and Injuries Collaborators.** "Global incidence, prevalence, years lived with disability (YLDs), disability-adjusted life-years (DALYs), and healthy life expectancy (HALE) for 371 diseases and injuries in 204 countries and territories and 811 subnational locations, 1990-2021." *Lancet.* 2024;403(10440):2133-61. — *Added to provide the most current Global Burden of Disease data on injury burden.*

- **References Retained with Updated Context:** The core references supporting our methodology (GOS-E Peds validation, REDCap, WHO injury definitions, HIMSS SDH framework) and key findings (trauma registries in Malawi and Ethiopia, pediatric trauma in LMICs) were retained. References were renumbered accordingly throughout the manuscript.
- **Summary:** The revised reference list contains 32 references, up from 31 in the original submission. All references have been verified as current and not retracted. The updates strengthen the manuscript by incorporating more recent evidence, particularly on food insecurity and health outcomes, while maintaining the foundational literature that supports our methodology and regional context.

3. **Consistency in reporting:** We have ensured consistency in terminology, variable labeling, and statistical reporting throughout the manuscript.

4. **Enhanced transparency:** We have added more detail regarding our analytical approach, assumption testing, and model selection to improve reproducibility.

**SUMMARY OF MAJOR CHANGES**

1. Corrected all p-values and ensured consistency with tables

2. Standardized terminology for "food insecurity" throughout

3. Relocated injury definition to the appropriate Methods subsection

4. Added reporting of assumption testing results

5. Clarified model selection rationale (modified Poisson vs. logistic regression)

6. Revised all table titles to use "mortality" instead of "non-survivor"

7. Corrected and clearly labeled all reference categories in regression tables

8. Addressed paradoxical food security finding with extensive discussion and appropriate citations

9. Revised Discussion opening paragraph to remove imprecise language

10. Substantially expanded the Limitations section to discuss how limitations affect measures of association

11. Processed all figures through PLOS NAAS tool

We believe these revisions have substantially strengthened the manuscript and addressed all reviewer concerns. We appreciate the opportunity to improve our work and hope the revised manuscript is now suitable for publication in PLOS Global Public Health.

Sincerely,

Natalie J. Tedford, MD, MScPH (Corresponding Author), on behalf of all co-authors
